# Supplementary material for: Microcystin-LR Removal from Water via Enzymatic Linearization and Ultrafiltration
Source: Toxins (Basel). 2022 Mar 22;14(4):231. doi: 10.3390/toxins14040231 (PMC9024530; doi:10.3390/toxins14040231)
Supplement: Supplementary file 1 [file toxins-14-00231-s001.zip › toxins-1605042-supplementary.pdf]

SupplementaryMaterials

Microcystin-LR Removal from Water via Enzymatic Linearization and Ultrafiltration

Abelline Fionah, Cannon Hackett, Hazim Aljewari, Laura Brady, Faisal Alqhtani, Isabel C. Escobar and Audie K. Thompson

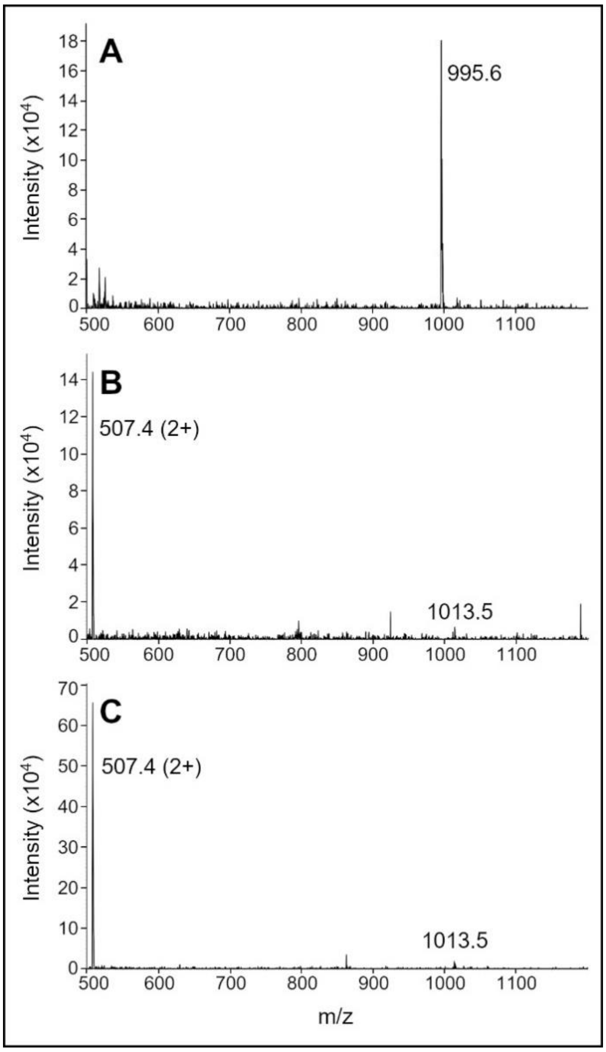

Figure S1. Mass spectrums of MC-LR solution as it is degraded by MlrA in an environment of phosphate buffer (5 mM), for samples taken 0 hours (5.03 min peak) (**A**), 4 hours (5.17 min peak) (**B**), and 24 hours (5.19 min peak) (**C**).

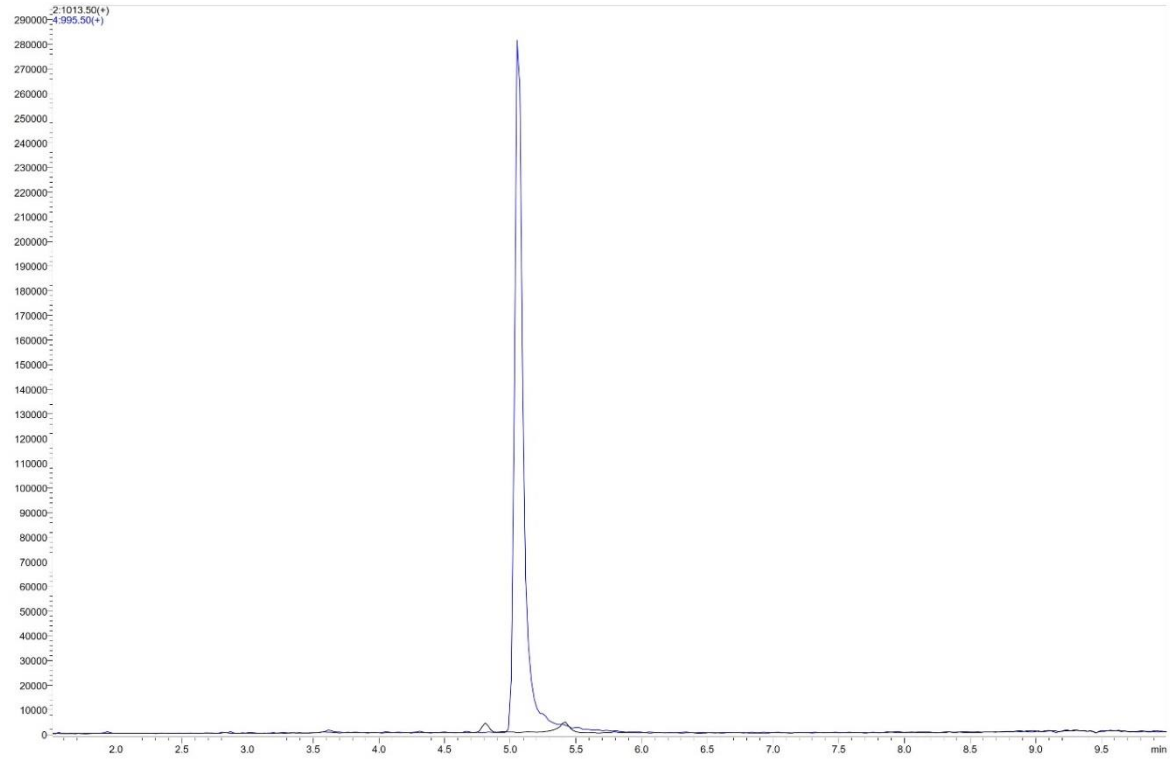

Figure S2. LC-MS SIM chromatogram for MC-LR as it is linearized by MlrA in an environment of phosphate buffer (5 mM), for the sample taken 0 hours after initial mixing (associated with Figure 2A). The curve for cyclic MC-LR (at *m*/*z* = 995.5) is shown in blue and the curve for linear MC-LR (*m*/*z* = 1013.5) is shown in black.

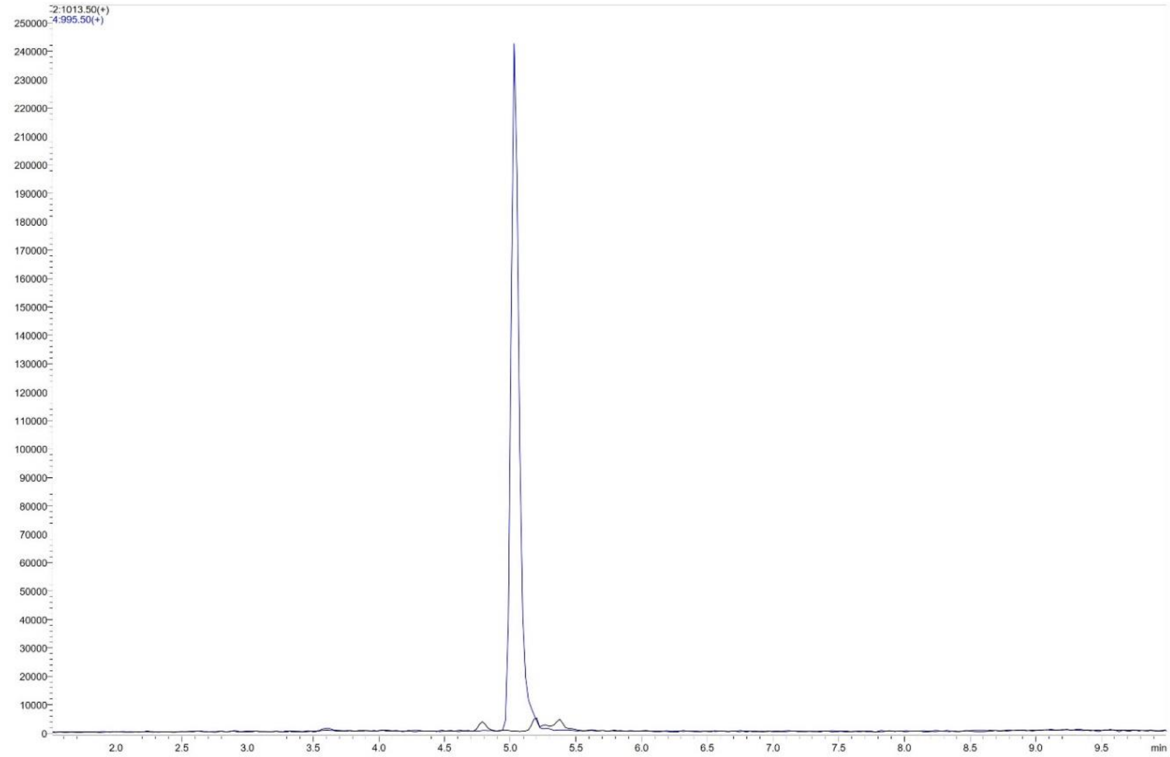

Figure S3. LC-MS SIM chromatogram for MC-LR as it is linearized by MlrA in an environment of phosphate buffer (5 mM), for the sample taken 4 hours after initial mixing (associated with Figure 2B). The curve for cyclic MC-LR (at *m*/*z* = 995.5) is shown in blue and the curve for linear MC-LR (*m*/*z* = 1013.5) is shown in black.

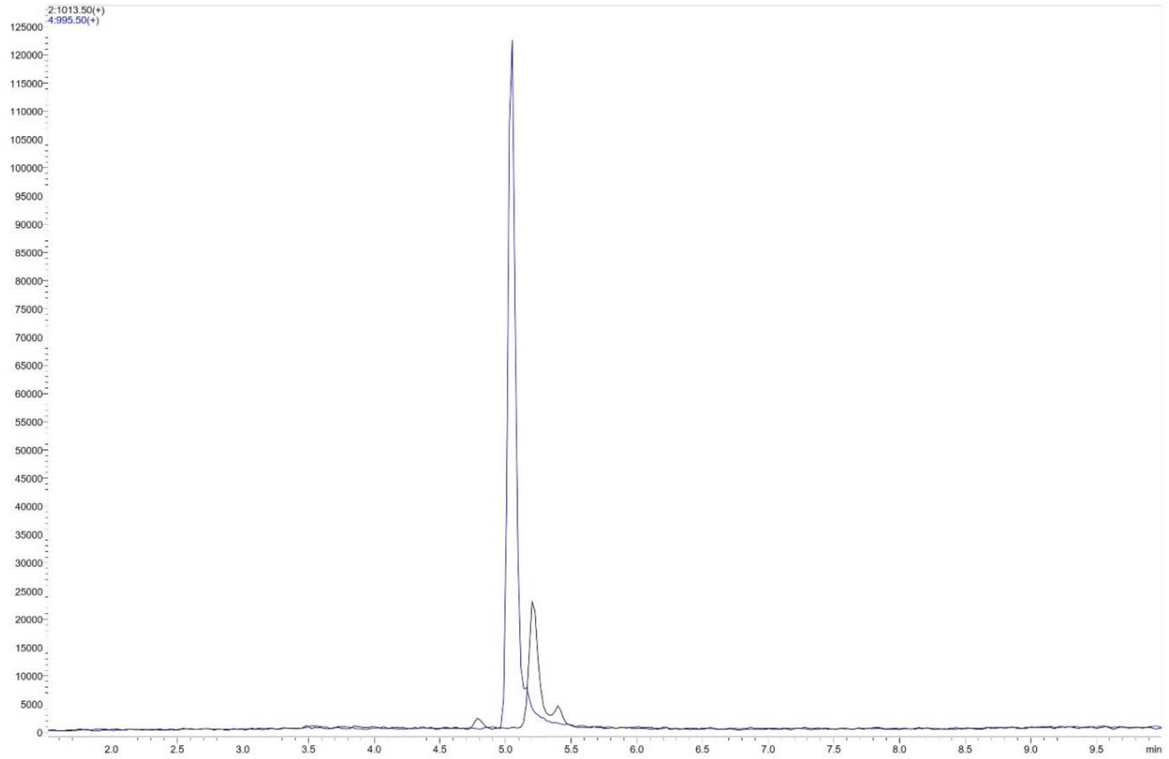

Figure S4. LC-MS SIM chromatogram for MC-LR as it is linearized by MlrA in an environment of phosphate buffer (5 mM), for the sample taken 24 hours after initial mixing (associated with Figure 2C). The curve for cyclic MC-LR (at *m*/*z* = 995.5) is shown in blue and the curve for linear MC-LR (*m*/*z* = 1013.5) is shown in black.

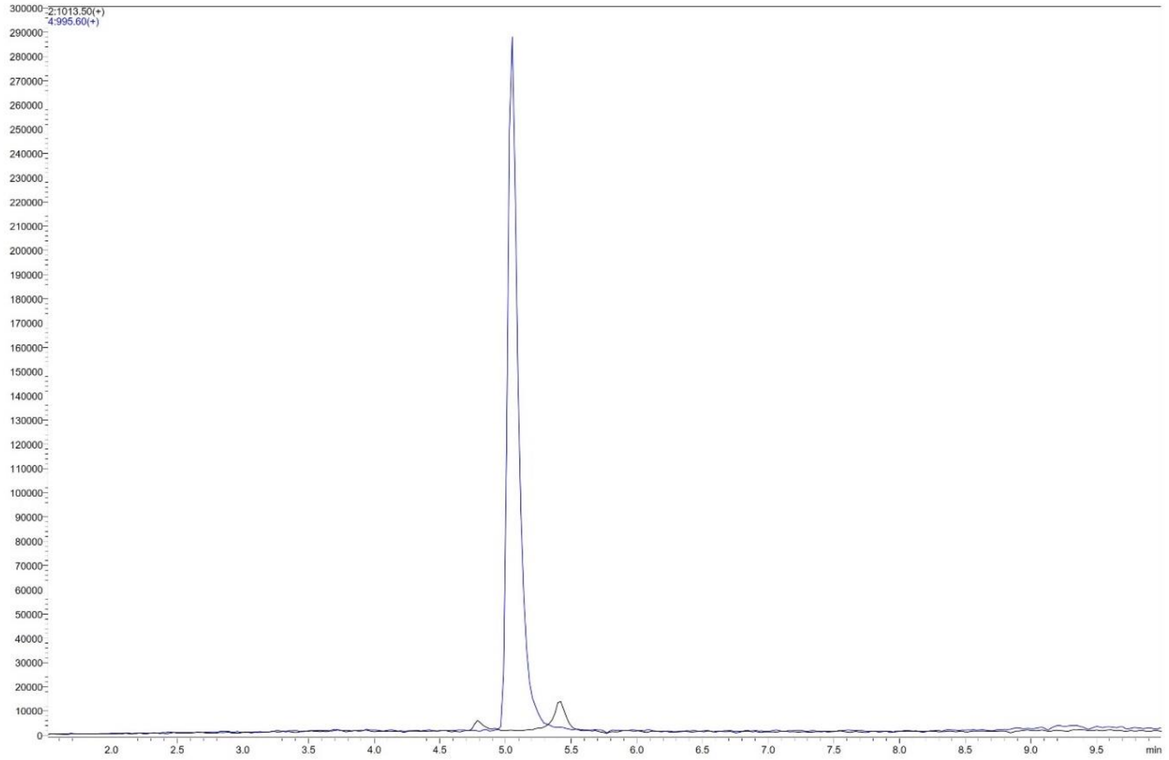

Figure S5. LC-MS SIM chromatogram for MC-LR solution incubated with a SPEEK-PSf membrane for the sample taken 0 hours after initial mixing (associated with Figure 5A). The curve for cyclic MC-LR (at *m*/*z* = 995.6) is shown in blue and the curve for linear MC-LR (*m*/*z* = 1013.5) is shown in black.

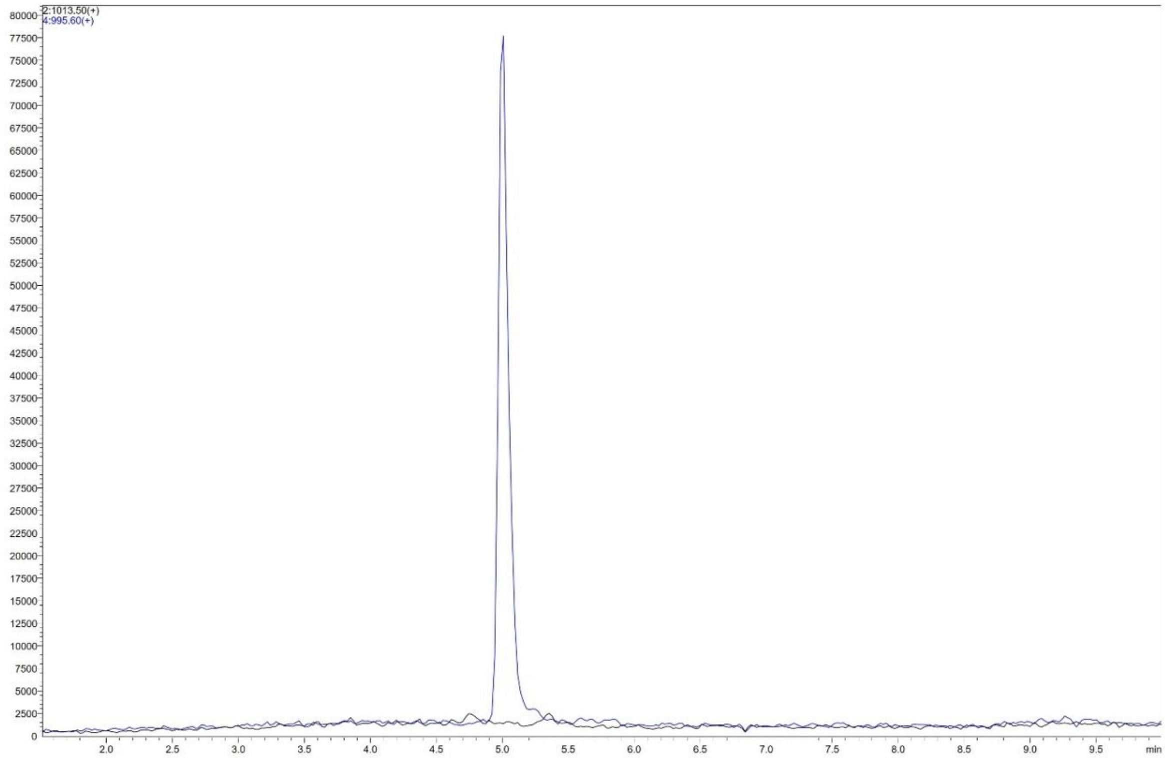

Figure S6. LC-MS SIM chromatogram for MC-LR solution incubated with a SPEEK-PSf membrane for the sample taken 4 hours after initial mixing (associated with Figure 5B). The curve for cyclic MC-LR (at *m*/*z* = 995.6) is shown in blue and the curve for linear MC-LR (*m*/*z* = 1013.5) is shown in black.

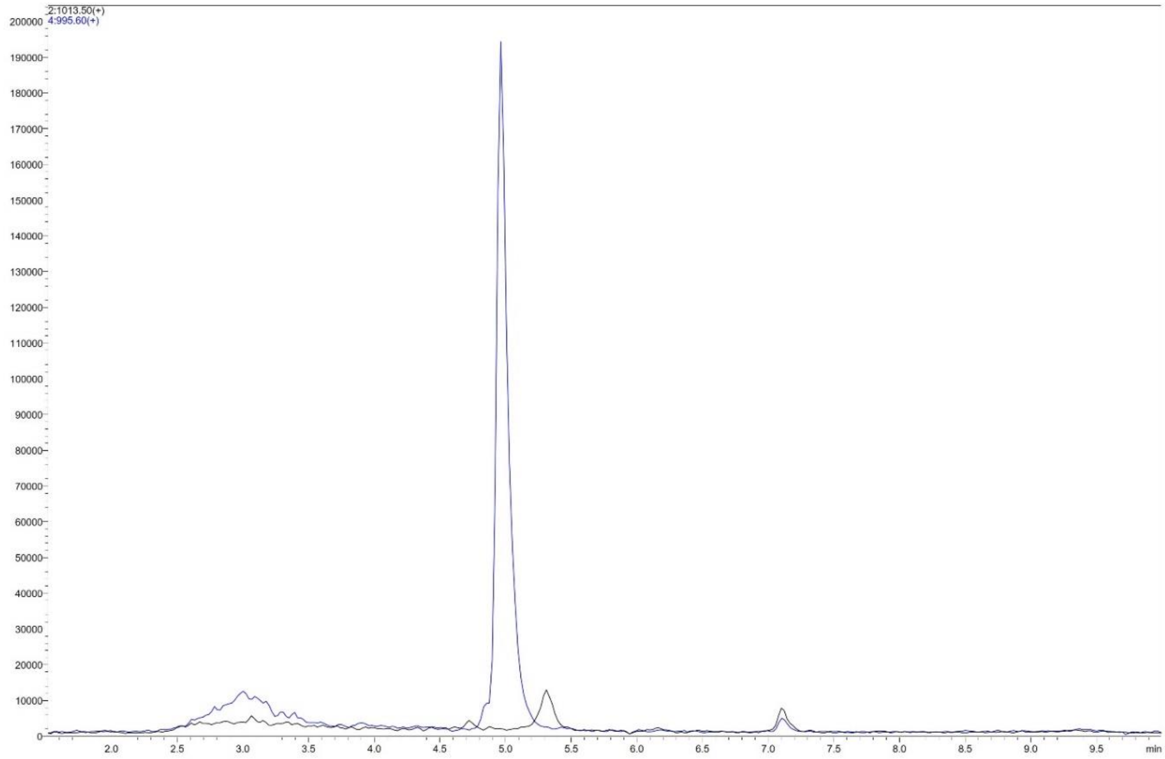

Figure S7. LC-MS SIM chromatogram for MC-LR solution incubated with a SPEEK-PSf membrane for the sample taken 24 hours after initial mixing and after adding methanol to cause desorption (associated with Figure 5C). The curve for cyclic MC-LR (at *m*/*z* = 995.6) is shown in blue and the curve for linear MC-LR (*m*/*z* = 1013.5) is shown in black.

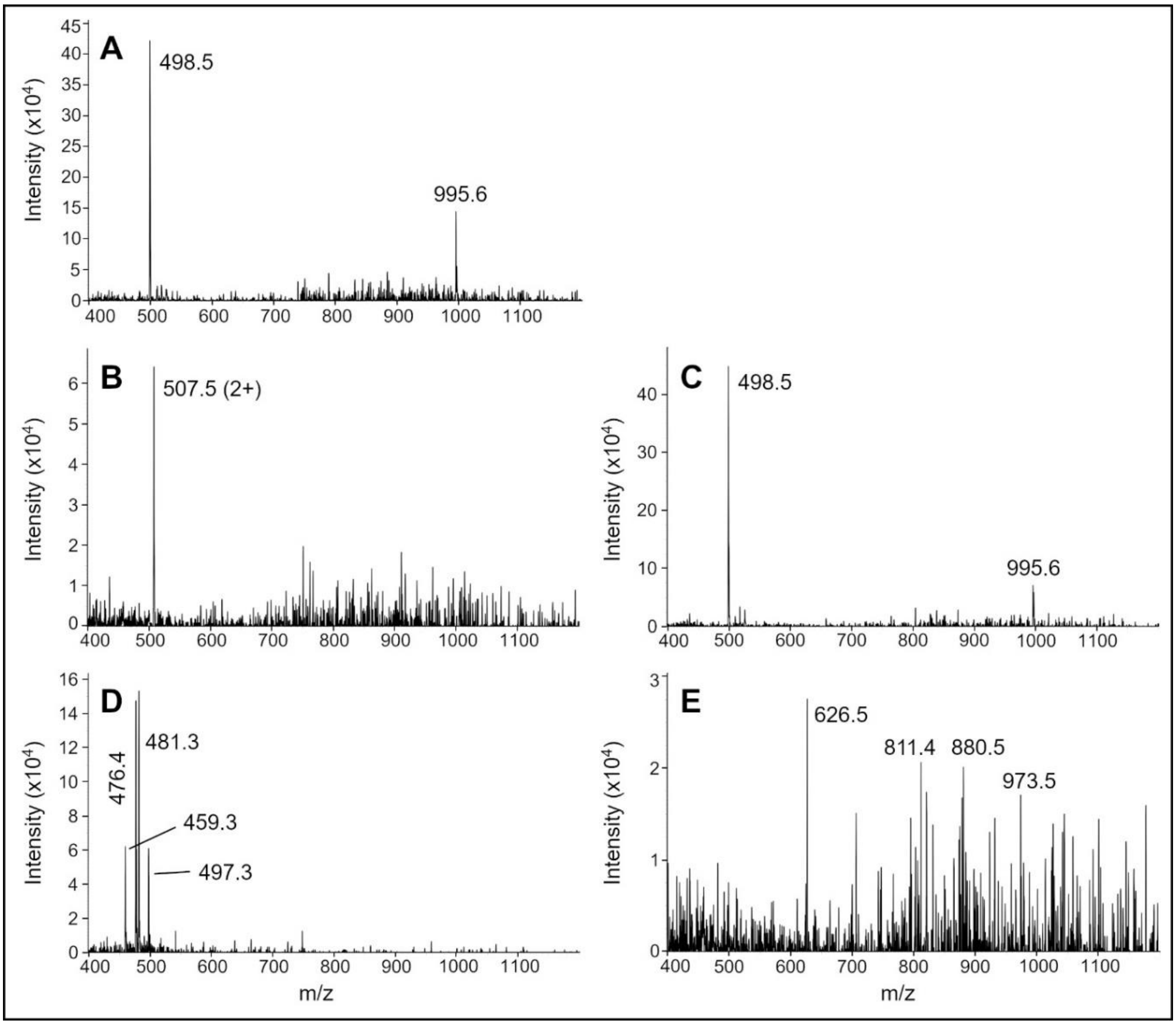

Figure S8. Mass spectrums of MC-LR solution incubated with MlrA, for samples taken at 0 hours (4.63 min peak) (**A**), 24 hours (4.09 min peak) (**B**), 24 hours (4.63 min peak) (**C**), and the solution filtered through a SPEEK-PSf membrane for the 1.73 min peak (**D**) and the 4.65 min peak (**E**).
